# Evaluating the Utility of the Joint Geriatric and Psychiatry Complex Parkinson’s Clinic: A Service Evaluation

**DOI:** 10.1192/bjo.2025.10476

**Published:** 2025-06-20

**Authors:** Tamara Chithiramohan, Kate O’Kelly, Lucy Beishon, Ann Boyle

**Affiliations:** 1 Leicestershire Partnership NHS Trust, Leicester, United Kingdom; 2 University Hospitals of Leicester, Leicester, United Kingdom; 3 University of Leicester, Leicester, United Kingdom

## Abstract

**Aims:** Patients with Parkinson’s disease commonly have comorbid mental health conditions. There is a direct interplay between Parkinsonian and antipsychotic medication which can lead to difficult management decisions. Most mental health and Parkinsonian services are separate, with little coordination or communication between services. A multidisciplinary (MDT) clinic in Leicester was started two years ago to allow an MDT approach to the care of complex Parkinson’s patients. This is held by a geriatrician, an old age psychiatrist, and a Parkinson’s specialist nurse. The aim of this service evaluation was to formally evaluate the utility of the clinic.

**Methods:** We gathered quantitative information from the medical notes of patients seen between November 2023–May 2024, and qualitative information via interviews with carers and Community mental health team (CMHT) psychiatrists. Demographic data was gathered, as well as whether the patient was under a CMHT, whether there was a psychiatric component to appointment, and have we avoided hospital admission. Carers and CMHT consultants were asked about their experience of the clinic and whether the joint service was helpful for them.

**Results:** Notes of 23 patients were reviewed. Most had a primary diagnosis of Parkinson’s disease and were on two or more psychiatric medications (69.6%) and Parkinsonian medications (69.6%). 43.5% were already known to CMHT. Most had a clear psychiatric input to each consultation, such as medication change, cognitive assessment, or cancellation of CMHT appointment. The medical notes suggested 30.4% avoided CMHT referral and 17.4% may have avoided hospital admission. Six of the 23 patients had a reduction in carer strain.

CMHT consultants felt it was a useful addition which could lead to more timely care of patients. Both felt they were not confident in managing complex Parkinson’s patients or adjusting Parkinsonian medication and would have to refer to either geriatrics, neurology or Parkinson’s specialist nurses.

We interviewed three carers of patients. All were very happy with the care received, felt it was superior to the separate care they received before and felt they received more holistic and timely care. All felt it had reduced carer strain and prevented admission to hospital.

**Conclusion:** Carers and patients have benefited from the joint geriatrics, old age psychiatry Complex Parkinson’s clinic, compared with the separate care they were receiving before. Clinicians and carers feel it has allowed for efficient and holistic treatment of patients, avoided further appointments, avoided hospital admission in some cases and reduced carer strain.

